# Responses to Induced Passive Heat in Two Local Common Bean (*Phaseolus vulgaris* L.) Varieties Under Humid Tropical Field Conditions in Costa Rica

**DOI:** 10.3390/plants14223489

**Published:** 2025-11-15

**Authors:** Idrissa Diédhiou, Josafath A. Otero, Oswaldo Navarrete, Yendry N. Arguedas-Flatts, Jorge Alonso Alcala Jauregui, Hugo M. Ramírez-Tobias

**Affiliations:** 1 Universidad EARTH, Las Mercedes de Guácimo, Guácimo 70602, Costa Rica; onavarrete@earth.ac.cr; 2 Escuela de Matemáticas, Universidad Nacional de Costa Rica, Campus Omar Dengo, C. Padre Royo, Heredia C.P. 86-3000, Costa Rica; yendry.arguedas.flatts@una.cr; 3 Facultad de Agronomía y Veterinaria, Universidad Autónoma de San Luis Potosí, Km. 14.5 Carretera San Luis-Matehuala, Apdo. Postal 32, Soledad de Graciano Sánchez C.P. 78321, San Luis Potosí, Mexico; jorge.alcala@uaslp.mx (J.A.A.J.); hugo.ramirez@uaslp.mx (H.M.R.-T.)

**Keywords:** Open Top Chamber (OTC), abiotic stress, high temperature, native seeds, *Matambú*, *Tayní*

## Abstract

Climate change is a major constraint for common bean (*Phaseolus vulgaris* L.) cultivation in tropical regions, where elevated temperatures drastically affect reproductive efficiency and yield. This study aimed to evaluate the response of two local varieties, *Matambú* and *Tayní*, under passive induced heat using Open Top Chambers (OTC) in the humid tropics of Costa Rica. A factorial randomized block design with two genotypes and two environments (control and OTC) was applied to assess morphological, physiological, and yield-related traits. OTC increased daily maximum, minimum, and mean air temperatures by +2.29, +0.93, and +2.80 °C, respectively, and raised cumulative growing degree days by 325 °C·day^−1^ compared with the control. Heat stress reduced grain yield by more than 80% (from 0.15 to 0.03 t·ha^−1^) and significantly lowered the harvest index, confirming strong reproductive vulnerability. However, *Matambú* maintained higher nodulation and above-ground biomass under heat, whereas *Tayní* showed marked declines in pod set and nodule number. Correlation analyses revealed that pod number and harvest index were the strongest predictors of yield across environments. These results provide the first field evidence of local varietal responses to induced passive heat in Costa Rican common bean varieties and highlight *Matambú* as a valuable genetic resource for breeding climate resilient cultivars.

## 1. Introduction

The escalating impact of climate change on global agriculture presents a critical threat to food security, particularly in low-latitude regions such as Central America. Among the most concerning trends are rising temperatures, an increased frequency of extreme heat events, and altered precipitation patterns [1,2]. For countries such as Costa Rica, where agriculture remains the backbone of rural livelihoods, there is an urgent need to develop crop systems that are resilient to these changes while maintaining productivity under increasingly unpredictable environmental conditions.

Common bean (*Phaseolus vulgaris* L.), a key source of protein and micronutrients for millions of people in Latin America and sub-Saharan Africa, is especially sensitive to elevated temperatures during its reproductive phase [3]. Heat stress above 30 °C can significantly reduce pollen viability, flower retention, and pod development, leading to yield losses of up to 80% in susceptible genotypes [4,5]. This situation is further aggravated by the prevalence of low-fertility soils, especially those deficient in phosphorus, an essential nutrient for root development and energy transfer [6,7].

Although genetic improvement programs have made significant strides in developing stress-resilient varieties, many commercial cultivars still exhibit limited adaptability to complex tropical environments. In contrast, common bean varieties, which are locally adapted varieties that have evolved over generations, often display unique combinations of physiological and agronomic traits that confer resilience under low-input and stress-prone systems [8,9]. Despite their potential to enhance climate adaptation, these genetic resources remain underutilized in formal breeding pipelines [10,11,12].

In Costa Rica, local varieties, such as *Matambú* and *Tayní*, continue to play a vital role in traditional farming systems. Preliminary evidence suggests that these local common bean varieties may possess adaptive traits to elevated temperatures and variable soil conditions [7,13], although empirical evaluation under controlled warming conditions has been limited [14,15]. Given the projected temperature increases for Central America, ranging between 1.5 °C and 3.0 °C over the next 50 years [2], there is a pressing need to test the thermal resilience of these genotypes in field-realistic scenarios.

The genotype × environment (G × E) interaction is a central framework for understanding how different genotypes respond to environmental variability. Investigating G × E under simulated climate change conditions can uncover not only which genotypes perform best under stress, but also why, revealing physiological processes and adaptive mechanisms. This knowledge is key for targeting specific traits in breeding programs and guiding policies for climate-resilient agriculture [16,17].

An experimental method that allows for realistic field-based simulation of climate warming is the use of Open Top Chambers (OTC). These passive heating structures increase the air temperature by trapping solar radiation, mimicking the predicted climate change without interfering with natural rainfall or soil properties [5,18]. OTC have proven effective in measuring the thermal impacts on crop phenology, photosynthesis, biomass allocation, and yield components across various crops [19,20], but their application remains limited in tropical legume research.

This study applied OTC technology in a tropical humid field setting to simulate projected climate warming in Costa Rica and evaluate the performance of *Matambú* and *Tayní* under induced passive heat. The variables of interest included temperature metrics (maximum, minimum, and mean), growing degree days (GDD), morphological traits (e.g., plant height and root length), reproductive traits (e.g., flower number and pod formation), yield and its components (e.g., grain weight and harvest index).

Understanding how local genotypes respond to warming scenarios will provide critical insights for both the conservation and genetic improvement of common bean germplasm. Moreover, identifying physiological and morphological traits associated with heat resilience may facilitate the targeted selection of climate-adapted varieties for the tropics supporting smallholder farmers in preserving agrobiodiversity while adapting to rising temperatures. In this context, the objective of this study was to evaluate the impact of induced passive heat on two local common bean genotypes in the tropical humid region of Costa Rica, using OTC to simulate field-relevant warming conditions. We hypothesized that the genotypes would exhibit differential morphological, physiological, and yield responses to elevated temperatures, reflecting inherent variability in thermal tolerance traits and G × E interactions.

## 2. Results

### 2.1. Abiotic Variables Under OTC and Control Plots

During the experimental period, the implementation of OTC significantly altered the thermal regime experienced by two local bean varieties under Costa Rica’s tropical humid conditions. The OTCs elevated critical temperature parameters throughout the crop cycle, closely matching climate projections for the region [2].

**Figure 1:**
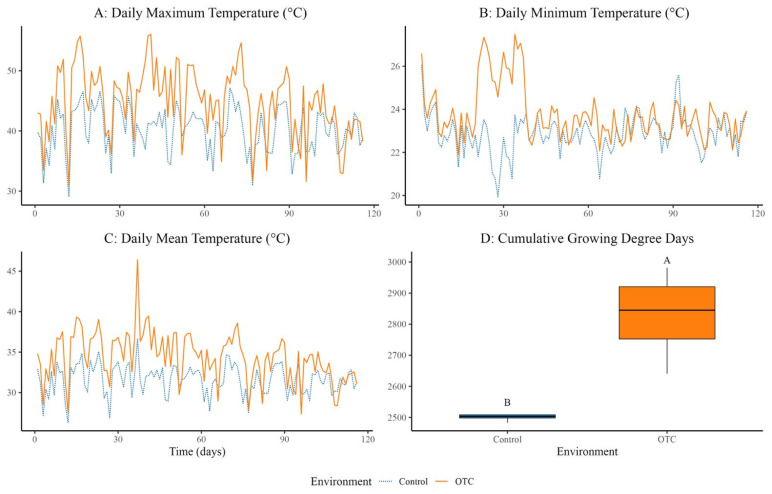
Abiotic variables recorded in control and OTC (Open Top Chamber) conditions during the experimental period. (**A**) Daily maximum temperature (°C), (**B**) daily minimum temperature (°C), (**C**) daily mean temperature (°C), and (**D**) cumulative growing degree days (°C·day^−1^) were monitored using SensorPush sensors installed in four replicated experimental units per environment (*n* = 4 per treatment). Different letters indicate significant differences at *p* < 0.05.

Daily maximum temperatures were significantly higher in OTC plots (42.52 ± 0.32 °C) compared to controls (40.23 ± 0.32 °C), reflecting an average warming of 2.29 °C (F = 52.33, MS = 1219.50, *p* < 0.0001) (Figure 1A). Daily minimum temperatures also increased, averaging 23.78 ± 0.10 °C in OTCs versus 22.85 ± 0.10 °C in controls (Δ = 0.93 °C; F = 41.28, MS = 49.617, *p* < 0.0001) (Figure 1B). The daily mean temperature rosed by 2.80 °C under OTC conditions, reaching 34.38 ± 0.23 °C versus 31.58 ± 0.23 °C in the control (F = 72.03, MS = 454.15, *p* < 0.0001) (Figure 1C). These temperature elevations created a consistent warming environment during both day and night (Figure 1).

Furthermore, cumulative Growing Degree Days (GDD) were significantly higher in the OTC treatment: 2828.1 ± 147.5 °C·day^−1^ compared to 2503.3 ± 16.6 °C·day^−1^ in controls (Δ = 324.8 °C·day^−1^; F = 19.16, MS = 210,976, *p* = 0.005), with distinct grouping by Tukey’s test (Figure 1D).

### 2.2. Effect of Genotype and Induced Passive Heating on Vegetative Growth and Development in Two Local Bean Varieties

Plant height, stem diameter, and number of leaves per plant (NLP) were the morphological variables for which no significant G × E interaction was found (Supplementary Data Table S1). Nonetheless, notable major impacts of both environment and genotype were noted. *Matambú* exceeded *Tayní* with regard of morphological performance across all genotypes. *Tayní* recorded 32.12 ± 0.05 cm, 3.00 ± 0.02 mm, and 5.85 ± 0.04 mean plant height, stem diameter, and NLP whereas *Matambú* recorded 47.18 ± 0.05 cm, 3.09 ± 0.02 mm, and 7.06 ± 0.04 mean plant height, respectively (Table 1).

Environmental factors also made a significant difference. Compared to plants exposed to higher temperatures in OTC, where the averages decreased to 36.73 ± 0.05 cm and 2.92 ± 0.02 mm, respectively, plants cultivated under ambient conditions (control) reached higher heights (41.26 ± 0.05 cm) and stem diameters (3.19 ± 0.02 mm). It’s interesting to note that the NLP in OTC conditions was greater (7.01 ± 0.04) than the control (5.89 ± 0.04), which may indicate a morphological adjustment under heat stress.

### 2.3. Effect of the Induced Passive Heat on Biomass Accumulation Variables in Two Local Varieties Bean

Significant effects of both genotype and environment were observed for root morphological traits, while no significant G × E interaction was detected.

*Matambú* demonstrated a considerably higher root fresh weight (5.97 ± 0.06 g) than *Tayní* (5.08 ± 0.06 g) in terms of genotype performance, indicating a larger allocation of biomass to belowground structures. Nonetheless, there was no statistical difference in root length between the two local varieties; *Matambú* and *Tayní* recorded 22.71 ± 0.05 cm and 21.63 ± 0.05 cm, respectively (Table 1).

Root development was significantly impacted by environmental factors linked to induced passive heat. In comparison to plants grown in ambient control conditions (4.57 ± 0.06 g and 20.81 ± 0.05 cm, respectively), plants grown in OTC under elevated temperature stress exhibited significantly larger root fresh weight (6.64 ± 0.06 g) and longer roots (23.60 ± 0.05 cm). This pattern points to a possible adaptive reaction to temperature stress, marked by accelerated root development, which could facilitate better water absorption or thermoregulation in adverse environmental circumstances (Table 1).

A common bean’s adaptive morphological response to temperature stress is suggested by the observed increases in root fresh weight and root length under induced passive heat settings. This increase in growth in hotter climates could be a tactic to increase soil moisture availability or lessen physiological stress brought on by heat. Interestingly, *Matambú* showed much more root biomass than *Tayní*, confirming its general morphological resilience in both environments. The absence of genotypic variations in root length suggests that while both types develop roots of comparable length, their allocation of biomass varies.

### 2.4. Effect of the Induced Passive Heat on Yield and Yield Components in the Two Local Varieties Bean

There was no significant G × E interaction for number of grains per pod (NGP), pod width, grain yield and Harvest Index (HI). However, there were clear individual impacts of environment and genotypes.

Although the difference was not statistically significant, *Matambú* produced a slightly more yield (0.11 ± 0.03 t ha^−1^) than *Tayní* (0.08 ± 0.03 t ha^−1^) in both environments. While the quantity of grains per pod remained statistically similar between the two local varieties (2.74 ± 0.11 and 2.43 ± 0.11, respectively), *Matambú* pods were noticeably broader (0.85 ± 0.04 cm) than *Tayní* (0.76 ± 0.03 cm) (Table 1).

The yield was significantly impacted by environmental factors. The productivity of plants cultivated in OTCs under high temperatures was significantly reduced; yield decreased to 0.03 ± 0.03 t ha^−1^ from 0.15 ± 0.03 t ha^−1^ under normal conditions. Temperature treatment had no effect on pod width; however, it did slightly decrease under OTC (0.77 ± 0.04 cm) in comparison to the control (0.84 ± 0.04 cm). With values of 2.64 ± 0.11 (OTC) and 2.51 ± 0.11 (control), the quantity of grains per pod stayed constant throughout environments.

These findings demonstrate how susceptible local bean yield is to climate change-simulating induced passive heat conditions. Yield formation is extremely susceptible to high temperatures in humid tropical environments like those found in Costa Rica, as seen by the clear decrease in productivity under temperature stress in the OTC an 80% decrease when compared to ambient conditions.

The substantial reduction in overall yield, despite the absence of significant differences in the number of grains per pod, suggests that temperature stress may have disrupted key reproductive processes such as pollination or pod filling. Although both genotypes maintained some level of productivity under induced passive heat, *Matambú* demonstrated more performance in yield and pod development compared to *Tayní*. These findings underscore the importance of advancing research and breeding efforts focused on bean cultivars with greater resilience to heat-induced environmental stress, as a strategic component of climate adaptation in tropical agriculture.

On the other hand, environmental factors had a considerable impact on the HI, whereas genotype had no effect. The mean HI of plants cultivated under control conditions was higher (0.23 ± 0.16), indicating a statistically significant decrease under raised temperature, than that of plants produced under OTC (0.07 ± 0.16). There was no difference between the genotypes of *Tayní* and *Matambú*, with average HI values of 0.12 ± 0.07 and 0.19 ± 0.07, respectively. These findings suggest that temperature stress significantly decreased the percentage of total biomass devoted to grain production, even while genotypic heterogeneity in HI was not significant.

The significant reduction in HI under induced passive heat conditions highlights a marked shift in biomass allocation away from reproductive output in both genotypes. Despite similar total biomass production across environments, plants exposed to elevated temperatures invested proportionally less in grain formation, leading to a lower HI. The absence of significant genotypic differences in HI suggests that both *Matambú* and *Tayní* were similarly affected in terms of reproductive efficiency relative to total biomass. These results highlight how common beans’ resource partitioning mechanisms are sensitive to temperature stress and highlight the significance of using HI as a crucial selection criterion in breeding initiatives aimed at producing climate resilient cultivars for tropical climates.

### 2.5. Effect of Induced Passive Heat on Reproductive and Biomass Traits of Two Local Bean Varieties

**Figure 2:**
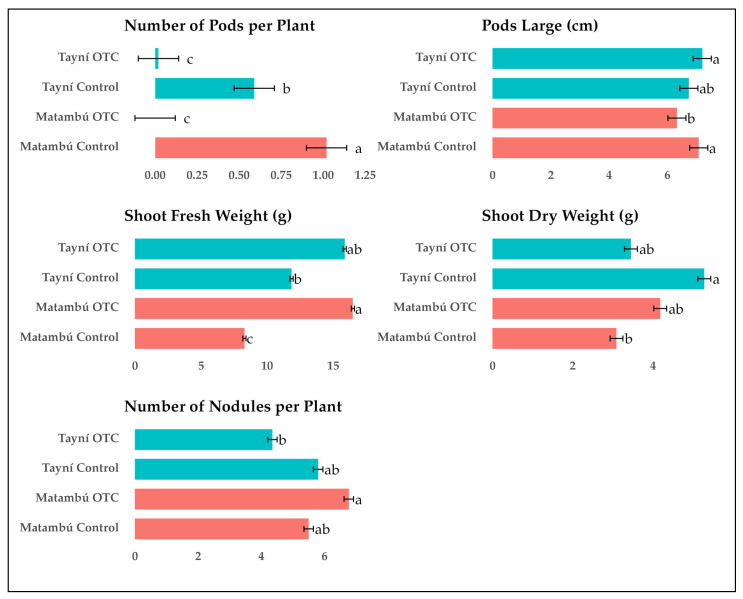
Interaction effects of Genotype and Environment on reproductive and biomass traits of two local bean varieties grown under ambient and induced passive heat conditions. *Tayní* and *Matambú* are the two local beans. *Different letters indicate statistically significant differences among Genotype × Environment interactions according to Tukey test (p < 0.05)*.

A highly significant interaction between G × E (Supplementary Data Table S1) was observed for all measured traits related to reproductive development and biomass accumulation, including number of pods per plant, pod length, shoot fresh weight, shoot dry weight, and number of nodules per plant (Figure 2). These results confirm that the response of both bean genotypes to induced passive heat was not uniform across traits and environments, indicating distinct genotype-dependent patterns of sensitivity and resilience to elevated temperature.

The number of pods per plant followed a similar interaction pattern. *Matambú* grown under control conditions produced the highest pod number (1.14), significantly greater than all other treatments. *Tayní* under control produced 0.56 pods per plant, while both genotypes under OTC conditions showed a strong reduction, with *Matambú* and *Tayní* recording only 0.17 and 0.14 pods per plant, respectively. This result indicates that although *Matambú* can maintain floral development under heat, it does not fully translate into pod set under stress, reflecting a reproductive bottleneck under elevated temperatures.

Pod length also varied significantly because of the G × E interaction. Under control conditions, *Matambú* produced the longest pods (6.38 cm), followed by *Tayní* (6.00 cm). Under OTC conditions, *Tayní* maintained relatively stable pod length (5.85 cm), while *Matambú* experienced a significant reduction to 5.56 cm. This pattern suggests that pod elongation in *Matambú* is more responsive and vulnerable to elevated temperature than in *Tayní*, which displayed lower pod length overall but less fluctuation between environments.

In terms of shoot fresh weight, *Matambú* under OTC conditions recorded the highest value among all treatments (16.17 g), significantly exceeding both its control counterpart (9.77 g) and all other combinations. *Tayní* under OTC and control conditions produced 14.27 g and 11.71 g, respectively. This unexpected result indicates that *Matambú* exhibited increased aboveground biomass accumulation under heat, possibly due to altered allocation patterns or increased water content.

Shoot dry weight showed a different trend. The highest dry matter accumulation was found in *Tayní* under control conditions (4.51 g), with *Matambú* under OTC and *Tayní* under OTC presenting intermediate values of 4.06 g and 3.90 g, respectively. *Matambú* under control showed the lowest shoot dry weight (3.30 g), suggesting that although shoot fresh mass increased under OTC in *Matambú*, it did not correspond to increased dry matter accumulation.

The number of nodules per plant also differed significantly across treatments. *Matambú* under OTC conditions recorded the highest number of nodules (5.93), followed by *Matambú* under control (5.51) and *Tayní* under control (5.41), with no significant difference between the two control treatments. The lowest nodule count was observed in *Tayní* under OTC conditions (4.12), representing a significant reduction in nodulation under heat stress for this genotype.

The results of this study provide clear evidence that *Matambú* exhibits greater resilience than *Tayní* when exposed to induced passive heat, particularly in traits related to reproductive potential and belowground symbiosis. While both genotypes experienced a decline in pod set under OTC conditions, *Matambú* maintained higher nodule formation under stress, and uniquely showed increased shoot fresh biomass under elevated temperature. In contrast, *Tayní* was notably more affected, with reductions in pod production, and nodule formation under heat stress in our field and experiment conditions.

These G × E interactions confirm that the impact of temperature stress on bean performance in tropical conditions is genotype-specific and trait-dependent. The ability of *Matambú* to sustain key growth and reproductive functions under heat stress suggests that this genotype possesses adaptive traits that confer greater tolerance to the anticipated conditions of climate change in humid tropical regions such as Costa Rica. These findings underscore the importance of evaluating multiple plant functions including reproductive development, biomass allocation, and root symbiosis when screening for climate-resilient bean cultivars. They also reinforce the necessity of integrated G × E assessments to identify stable performers for future climate scenarios.

### 2.6. Leaf Physiological Nutrient Content Under Induced Passive Heat (OTC)

**Figure 3:**
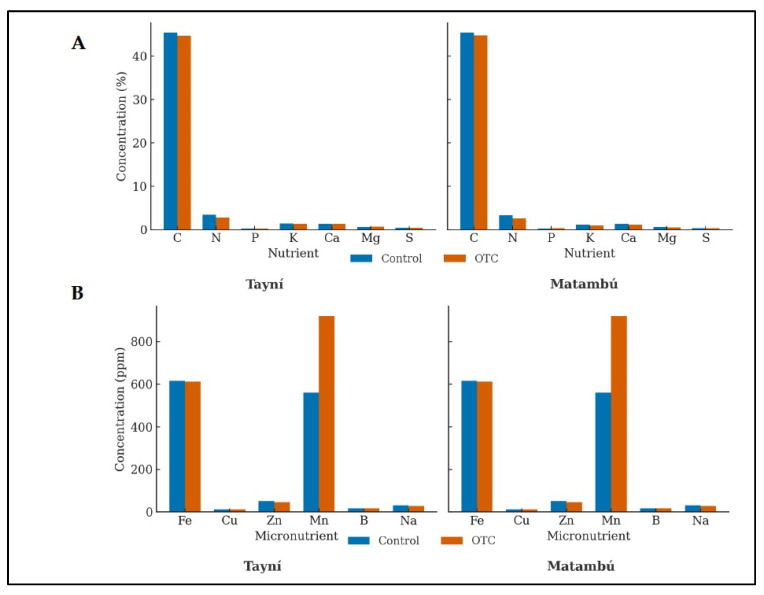
Effect of induced passive heat on leaves macronutrient (**A**) and micronutrient (**B**) concentrations of *Tayní* and *Matambú* bean varieties. *Tayní* and *Matambú* are the two local beans used in the experiment. OTC: Open Top Chamber.

The foliar nutrient profile revealed clear differences between genotypes and temperature treatments (Figure 3). Overall, both genotypes showed similar macronutrient composition, with C and N representing the dominant fractions. Under OTC conditions, a marked decline in leaves N concentration was observed in both *Tayní* and *Matambú*, suggesting a heat induced inhibition of N assimilation or dilution due to changes in biomass accumulation. In contrast, the concentrations of K, Ca, Mg, and S remained relatively stable across treatments, indicating that macronutrient homeostasis was largely maintained despite thermal stress.

Micronutrient patterns displayed a stronger treatment effect. Under OTC conditions, both genotypes exhibited a notable increase in foliar Mn concentration, particularly pronounced in *Matambú*, which recorded almost a double rise compared with its control counterpart. This enrichment could reflect an adaptive mechanism related to antioxidant defense, as Mn plays a key role in the detoxification of reactive oxygen species. Fe concentrations remained high and stable, while Zn and Cu showed only minor fluctuations, suggesting that their uptake and translocation were not substantially altered by elevated temperature. The consistency in B and Na contents across treatments also supports the stability of micronutrient allocation under heat stress.

Collectively, these results indicate that induced passive heat did not drastically disrupt leaf nutrient composition but selectively enhanced Mn accumulation for *Tayní* and *Matambú*.

### 2.7. Pearson Correlation Analysis Among Agronomic and Environmental Variables

The Pearson correlation analysis showed that there were multiple important links between the thermal, morphological, physiological, and yield related variables in both temperature treatments. As expected, there were significant positive correlations between the three temperature variables: daily maximum temperature (Tmax), minimum temperature (Tmin), and mean temperature (Tmean). The coefficients were greater than r = 0.85 (*p* < 0.01), which confirmed that the thermal trends were consistent throughout the experiment (Table 2).

In addition, vegetative traits, including plant height (PH), stem diameter (PS), and number of leaves per plant (NLP), showed significant positive correlations (*r* = 0.60–0.75), suggesting coordinated shoot development under both environments. Shoot fresh weight (SFW) and shoot dry weight (SDW) were also strongly correlated (*r* > 0.80), confirming that biomass accumulation was consistent in both water and dry matter components.

Different behavior was shown by root traits. Interestingly, there was a substantial negative connection between root length (RL) and root fresh weight (RFW) (r = −0.62, *p* < 0.01), suggesting that biomass was not positively correlated with longer root systems. This pattern was present across both environments and suggests differences in root architecture or allocation strategy between genotypes or treatments, such as increased elongation at the expense of radial development.

On the other hand, there were considerable internal connections with reproductive features. There was a substantial link between harvest index (HI), number of pods per plant (NPoP), and pod weight (PW). The strongest correlation was found between NPoP and HI (r > 0.70, *p* < 0.01). Additionally, there were favorable correlations between final yield indicators and pod length (PL) and number of grains per pod (NGP).

Minimum temperature (Tmin) and mean temperature (Tmean) demonstrated moderately negative correlations with yield related traits like HI and NPoP. For the variable yield, the results showed a negative and very high correlation with the abiotic variables (Tmax, Tmin and Tmean) with a coefficient of −0.98 and these results indicated that temperatures especially at night may have affected reproductive efficiency in both environments, even though temperature variables were generally not strongly correlated with vegetative growth.

In summary, the correlation patterns observed across environments highlight the interconnected nature of vegetative growth, root development, and reproductive success in common bean. Traits such as pod number, pod weight, and HI were closely aligned with yield outcomes and may serve as useful indicators for selecting genotypes with consistent performance under both ambient.

## 3. Discussion

### 3.1. Simulation of the Passive Induced Heat with the Use of OTC

The OTC treatment effectively replicated the warming levels anticipated for Central America through daily temperature increases [2]. In line with anticipated mid-century warming predictions, average maximum, minimum, and mean temperatures increased by +2.29 °C, +0.93 °C, and +2.80 °C, respectively. Other OTC investigations, which frequently report temperature rises of 1–3 °C, are consistent with this level of warming [5,18,21]. Furthermore, the fact that cumulative Growing Degree Days (GDD) under high temperatures increased by an extra 324.8 °C·day^−1^ shows how useful OTCs are for speeding up development and simulating prolonged heat exposure.

Heat stress and crop phenological acceleration are commonly measured using such increases in GDD [22]. Long-term exposure to temperatures frequently results in reproductive failure for common beans, especially when mean or nocturnal temperatures surpass 30 °C. OTCs offer a reliable platform for assessing G × E responses in common beans by simulating these conditions, especially when heat loads are comparable to those predicted under SSP2-4.5 climate scenarios [7,23].

Our results support the notion that OTC-based thermal treatments provide a reliable, applicable simulation of near future warming conditions, which makes them appropriate for evaluating tropical legume resistance. Finding heat-adapted genotypes will require future research aimed at demonstrating phenological and yield responses under this degree of thermal elevation.

### 3.2. Principal Influence of Induced Passive Heat and Genotype

The absence of G × E interaction for some vegetative, root, and yield parameters in the two local bean landraces (*Tayní* and *Matambú*) indicates that both genotypes reacted to high temperatures in a similar way. The significant effects of environment and genotype, however, highlight how important these factors are to plant performance under passive induced heat stress.

Regardless of the environment, *Matambú* consistently surpassed *Tayní* in terms of plant height, stem diameter, and number of leaves per plant. Both genotypes showed increased leaf number but decreased height and stem thickness with OTC warming, indicating a compensatory morphological adaptation. Our results are consistent with those of [1], who found that *Phaseolus* genotypes that are under heat stress produced more leaves in hotter conditions, possibly improving canopy function and thermoregulation.

In addition, in both conditions, *Matambú* produced a significantly higher root fresh weight than *Tayní*. Additionally, plants treated to OTC showed a considerable increase in root length and fresh weight. This root improvement in response to heat stress is consistent with research on other legumes, where higher belowground allocation supports physiological balance and water intake under hotter conditions [24,25,26].

Under induced heat stress, grain yield drastically decreased. In the OTC environment, it decreased by more than 80%, from 0.15 t·ha^−1^ under control conditions to just 0.03 t·ha^−1^ with passive warming. Our results are consistent with the milpa-system study conducted in southern Mexico by [5], where in common beans interplanted with maize and squash under comparable OTC-based warming regimes showed a significant delay in yield formation, even though their vegetative development was improved. Despite an increase in early-stage biomass, their study found that passive heating dramatically reduced bean production, suggesting that reproductive activities are disproportionately affected by thermal stress in comparison to vegetative development.

In addition, Harvest Index (HI) was slightly greater in *Matambú* than in *Tayní*, although this difference was not statistically significant. Common beans and other legumes have also shown comparable harvest index decreases because of thermal stress, highlighting the importance of HI as a phenotypic selection target in climate-adaptive breeding techniques [27].

Our results further support the view that heat stress compromises reproductive efficiency more than vegetative growth, emphasizing that yield components and harvest index must be prioritized in breeding programs for climate resilience.

In sum, *Matambú* consistently performed better than *Tayní* in vegetative and root traits, paired with modestly more yield and pod morphology. The no interaction G × E suggests that these genotypic differences are constitutive rather than environment specific. Elevated leaf number and root growth under heat seem to be adaptive plastic responses. Consequently, traits such as root biomass, leaf plasticity, and harvest index emerge as promising targets for breeding programs aiming to enhance heat resilience in tropical bean systems.

Our results strongly align with research highlighting the importance of vegetative and root traits in heat tolerance selection [23,28]. The consistent performance of *Matambú* under both ambient and elevated temperatures suggests that traditional landraces may harbor inherent resilience traits adapted to tropical lowland conditions a finding that supports their strategic incorporation into climate-smart breeding pipelines.

These findings demonstrate that morphological development in local varieties of beans may be modified in variety-specific ways even under brief exposure to elevated temperatures, as anticipated under climate change scenarios. Under heat stress characteristic of Costa Rica’s tropical humid lowlands, the rise in NLP under thermal stress, especially in the more resilient *Matambú*, may be an early compensatory mechanism that aids leaf-based cooling or improves light interception. The significance of choosing and encouraging heat-resilient genotypes as part of climate adaptation methods in tropical bean production systems is highlighted by the inconsistent varietal response.

### 3.3. Significative Interaction G × E Under Induced Passive Heat

This study revealed strong G × E interactions for some key reproductive and biomass traits under induced passive heat in two local beans, *Matambú* and *Tayní*, grown in Costa Rica’s tropical humid. These interaction patterns highlight distinct genotype specific sensitivities and adaptive responses to elevated temperatures.

The patterns of interactions showed that the impact of warming is trait-dependent and heavily impacted by genetic background, rather than being consistent across genotypes. *Tayní* significant drop in flower production under OTC conditions demonstrated how sensitive reproductive initiation was, but *Matambú* levels remained similar to the control.

Changes in pollen viability and flower retention under heat stress have been linked to similar genotype-dependent responses in floral development [29]. However, under high temperatures, both genotypes showed significant reductions in pod set, demonstrating that flower production alone is not sufficient for successful reproduction when fertilization and early embryo development are impaired. This phenomenon has been repeatedly observed in common beans under heat stress [30].

Pod morphology also revealed individual strategies where *Tayní* maintained stable, but basically smaller, pods under heat stress, whereas *Matambú* longer pods under control conditions were drastically reduced. Although at a baseline lower performance level, such stability may be a sign of morphological resilience. Our findings are in concordance with [31] who stated that pod size stability under stress might be an adaptive strategy to ensure optimum seed development instead of optimizing yield potential, permitting certain genotypes to protect their reproductive performance under adverse conditions and act as a defense against environmental variations.

On the other hand, biomass accumulation patterns diverged as well. *Matambú* displayed an unexpected increase in shoot fresh weight under OTC, yet without a corresponding rise in dry matter, implying elevated tissue hydration rather than structural growth. In contrast, under heat stress, *Tayní* highest dry biomass under control conditions did not adapt. Trait-based heat response studies in *Phaseolus* species, which show biomass, chlorophyll, and PSII efficiency as powerful markers of thermal resilience, are consistent with these findings as stated by [5,18].

In addition, root nodulation showed clear expansions of G × E effects. *Tayní* showed notable reduction, whereas *Matambú* either preserved or increased nodule development under heat. Due to the decreases in nodule functionality and nitrogen fixation, the legume-rhizobia symbiosis is extremely susceptible to high temperatures [32], *Matambú* stability in this trait speaks to underlying adaptive potential that could support nutrient acquisition under climate stress.

As stated by [33] theses contrasting responses mirror recent evidence that heat tolerance in *P. vulgaris* is expressed through the stability of multiple physiological processes, particularly reproductive success and nitrogen fixation. In our case, the genotypes *Matambú* and *Tayní* responded differently in one to passive induced heat where *Matambú* demonstrated greater resilience, sustaining flower production, maintaining nodulation, and accumulating fresh biomass under passive heat, whereas *Tayní* showed marked reductions in floral initiation, pod set, and nodulation. In addition, finding genotypes like *Matambú* that maintain vital functions under stress will be crucial for breeding programs and for maintaining bean production systems in Central America as heat waves increase in tropical humid regions [2].

### 3.4. Effect of the Induced Passive Heat on Physiological Content of the Local Beans Varieties

In our study, the observed decline in foliar nitrogen (N) concentration under the OTC treatment in both genotypes supports the notion that elevated temperature interferes with N assimilation or induces a dilution effect via increased biomass or altered partitioning. Such disruption of nitrogen metabolism under heat stress has been documented in multiple crop systems, where high temperature reduces nitrate reductase activity and compromises root nutrient uptake and remobilization processes [34]. Additionally, a recent review emphasizes that optimal N nutrition enhances thermotolerance, given its central role in photosynthetic apparatus maintenance and stress responsive metabolites [35].

The relative stability of other macronutrients (K, Ca, Mg, S) observed in our data suggests that plants may preferentially maintain ionic homeostasis of structural and osmotic regulators, even under heat stress consistent with the idea that the nutrient disruption induced by heat is selective rather than wholesale [36]. The considerable genotype specific increase of foliar Mn under OTC conditions suggests a suspected adaptation mechanism linked to micronutrient control during heat stress. As stated by [37] Mn Manganese plays essential roles as a cofactor in superoxide dismutase (Mn-SOD) activity and the oxygen-evolving complex of photosystem II, thereby contributing to oxidative stress mitigation and photosynthetic resilience. In addition, Mn concentrations under heat stress, therefore, may reflect a stress induced mobilization or redistribution of Mn into protective pathways. In contrast, the minimal changes in Fe, Zn, Cu, B and Na further support the view that micronutrient responses under heat are element specific and tied to functional demand rather than passive accumulation.

### 3.5. Correlation Patterns Among Thermal, Morphological, and Yield-Related Traits

The consistency of induced passive heating across contexts is confirmed by the high positive correlations between thermal variables (Tmax, Tmin, and Tmean). More significantly, the strong correlations found between reproductive, physiological, and morphological parameters demonstrate how effectively coordinated mechanisms control yield performance under stress. Positive correlations between vegetative parameters including plant height, stem diameter, and leaf number support the idea that shoot development in beans and other legumes is closely regulated [38,39]. Similarly, aboveground development under both conditions appears to have followed identical allocation procedures, as indicated by the significant correlation between shoot fresh and dry biomass.

On the other hand, root characteristics showed a different pattern: a negative correlation between root length and fresh weight indicated that elongation occurred at the expense of radial thickness. Given that lengthened but thinner roots can enhance exploration in drying soils while compromising storage capacity, this trade-off may represent adaptive flexibility to heat stress [40,41]. As traditional selection based only on aboveground biomass may miss important stress-adaptive features, such architectural modifications highlight the necessity of including belowground qualities in climate-resilience breeding.

The reproductive domain revealed the strongest functional correlations. Pod number, pod weight, and harvest index were tightly linked to yield, with pod number emerging as the best predictor (r > 0.70). These results agree with [7,30], who showed that pod set stability is a decisive determinant of heat resilience in *Phaseolus vulgaris*. Furthermore, even minimal night warming can significantly decrease reproductive efficiency, as has been widely documented for beans and other legumes under climate change scenarios. This is confirmed by the strong negative associations between minimum/mean temperature and yield traits.

Collectively, these correlation patterns emphasize that yield resilience in beans depends on the integration of multiple functional aspects. Pod set and harvest index stand out as robust indicators for heat tolerance, while root and biomass traits reveal adaptive limitations that require careful consideration. These insights support a multidimensional approach to breeding those accounts for both aboveground and belowground processes in evaluating climate resilience of local germplasm.

This study represents an initial stage in the broader assessment of heat tolerance and physiological responses of native *Phaseolus vulgaris* germplasm from Costa Rica. The current evaluation, limited to two contrasting landraces (*Tayní* and *Matambú*) under induced passive heat and control conditions, provides baseline evidence of variation in nutrient composition and physiological performance. Future stages of this research will expand the analysis to a wider range of genotypes and agroecological origins to achieve a more inclusive and robust understanding of thermal resilience in local bean populations.

## 4. Materials and Methods

### 4.1. Experimental Establishment, Design, and Agronomic Practices

This study was carried out at EARTH University (Costa Rica). The geographical coordinates of the locality are 10°13′00.0″ N 83°35′27.0″ W with 43 m above sea level (m a.s.l.) The geographical area corresponds to the tropical and humid region of Costa Rica with more than 3701.99 mm of precipitation per year and an average of 25 °C of temperature with 86% of humidity [42].

**Figure 4:**
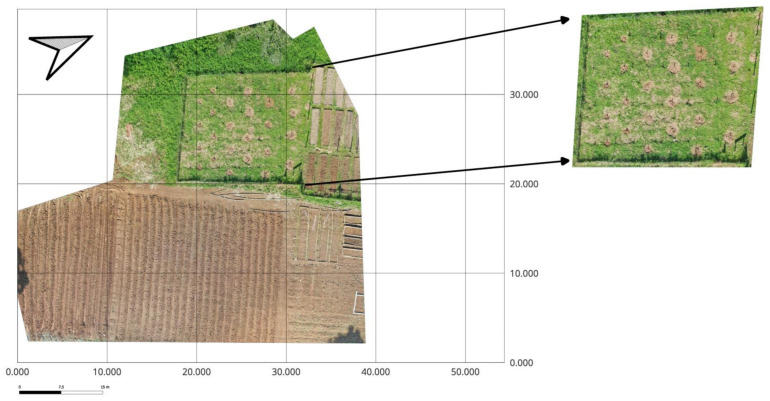
Aerial orthomosaic of the experimental field at EARTH University (Guácimo, Costa Rica) showing the study area (**left**) and a zoomed view of the bean parcel (**right**). The parcel hosted control plots and Open Top chambers (OTCs) used to induce passive warming. North arrow and scale bar are provided for orientation. Inset created on 3 February 2025 (image by Daniela Ramírez).

The study was conducted from July to October 2024 using a factorial Randomized Block Design. The first factor was local bean varieties (*Matambú* and *Tayní*), and the second factor was represented by the environment [passive induced heat with the use of the (OTC) and control], with six replications. The experiment included a total of 12 plots of 1.73 m^2^ each, and a total of 24 plots for the whole experiment (2 × 2 × 6) (Figure 4). The local bean varieties are widely cultivated by Indigenous and rural communities in the southern Caribbean region due to their adaptation to humid tropical climates and cultural significance. Before seeding, germination experiments were made to test the viability of the local seeds of beans. Those experiments were made according to the International Seed Test Association (ISTA) [43], where the seeds revealed up to 70% of the percentage of germination.

Agronomic practices and plant protection measures (daily irrigation and elimination of undesirable plants were accomplished throughout the crop’s growth period. Irrigation was undertaken immediately after sowing.

Before the establishment of the experiment, composite soil samples (0–20 cm depth) were collected from the experimental plots to determine basic physicochemical properties. The soil was moderately acidic (pH 4.79) with an extractable acidity of 1.9 cmol(+)/kg. Exchangeable bases were 2.73, 0.88, 0.23, and 0.74 cmol(+)/kg for Ca^2+^, Mg^2+^, K^+^, and Na^+^, respectively. Available phosphorus was low (1.51 mg/kg), while micronutrients such as Fe (68.6 mg/kg), Mn (45.4 mg/kg), and S (65.4 mg/kg) were within adequate ranges. The soil contained 2.03% total carbon and 0.16% total nitrogen, corresponding to a C/N ratio of approximately 12.7 (Supplementary Data Table S2). Overall, the soil exhibited the characteristics of a moderately acidic tropical soil with low base saturation and limited available phosphorus, typical of humid lowland environments in Costa Rica.

### 4.2. Simulation of the Induced Passive Heat

**Figure 5:**
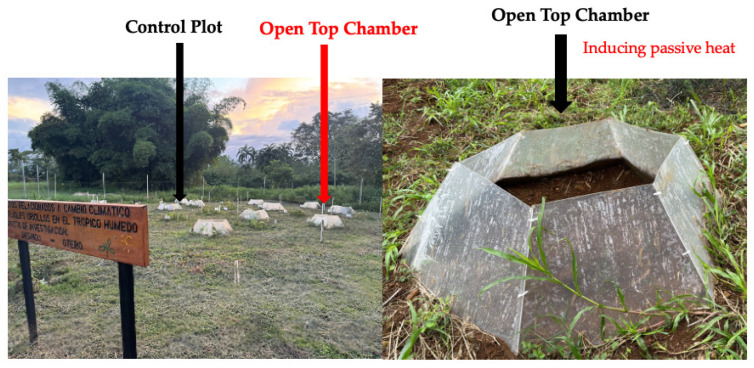
Experimental site at EARTH University (Guácimo, Costa Rica) showing the control plot (**left**) and the Open Top Chambers (OTC) used to induce passive heat (**right**).

Open top chamber (OTC) structures were used to simulate the induced passive heat. These structures allow for passive heating and are a simple method for monitoring plant responses to abiotic variables such as temperature increases in the field. The OTC were constructed using UV-resistant transparent acrylic (3 mm thick; wavelength transmission 110 < 280 nm) in accordance with [19,44]. The finished structures were 0.8 m tall, 1.5 m wide at the open top, and 2.08 m wide at the surface base (Figure 5). When compared to external ambient circumstances, this OTC design raises the air temperature by 1.9 to 5.0 °C during the day [5,18,45]. Across the experiment, the magnitude with which OTC altered the microclimate (air temperature) was regularly recorded both within and outside these structures.

### 4.3. Abiotic Variables Measurement

Temperatures were registered with data-loggers SensorPush (HT. w sensor manufacturer by Cousins/Sears LLC 2017, New York, NY, USA). Each OTC and control plot had one data logger mounted 10 cm above the ground in the center. The readings were scheduled to be taken every hour and averaged daily. These measurements were taken from July to October 2024, and the daily mean, minimum, and maximum air temperatures in each environment were calculated using the recorded data (OTCs and control). With the daily mean air temperature, the daily accumulated heat units were calculated with the residual classic method, which uses the following expression [46,47].

Cumulative Growing Degree Days=DMT−Tb

where: DMT = Daily Mean Temperature

Tb: Temperature base for beans (10 °C)

In addition, the sums of the daily accumulated heat units during all the experiment were used to determine the Cumulative Growing Degree Days (GDD) [48] for each SensorPush in each environment and were compared between the two treatments.

### 4.4. Agronomic Variables Measurement

#### 4.4.1. Vegetative Growth and Development Variables Measurement

4.4.1.1. Plant Height and stem

In each environment (OTC and control); 5 plants were selected for a total of 30 plants which were used to determine the plant height and stem from 10 days after seedings (das) in each experimental unit to 52 das.

4.4.1.2. Number of leaves per plant

The number of leaves was determined counting the number of leaves of 5 plants in each experimental unit for a total of 60 plants in all the experiment. The counting was made 45 days after seedings.

#### 4.4.2. Biomass Accumulation Variables

Vegetative and root development traits were assessed at the flowering stage on 18 plants per genotype and environmental treatment, totaling 72 observations. The following variables were measured:

4.4.2.1. Shoot fresh weight (g): the aboveground biomass was cut at the soil surface and immediately weighed.

4.4.2.2. Shoot dry weight (g): samples were oven-dried at 65 °C until constant weight and recorded using a digital scale.

4.4.2.3. Root fresh weight (g): roots were carefully washed and blotted dry before weighing.

4.4.2.4. Root length (cm): the primary root length was measured from the root crown to the tip using a ruler.

#### 4.4.3. Symbiotic Function (Nitrogen-Fixation Potential)

4.4.3.1. Number of nodules per plant: nodules were manually counted after gently washing the root system. Also, 5 plants were used for each genotype and environment for a total of 60 plants.

4.4.3.2. Yield and yield Components Variables Measurement

Yield component measurements were carried out on 20 mature pods randomly selected per genotype and per environmental treatment (Control and OTC), resulting in a total of 40 pods per genotype. For each pod, three variables were recorded:

4.4.3.2.1. Pod width (cm): measured at the widest point using a precision digital caliper.

4.4.3.2.2. Pod length (cm): measured from base to tip using a digital ruler.

4.4.3.2.3. Number of grains per pod: obtained by manually counting the seeds inside each pod.

These data were used to evaluate the influence of genotype and thermal environment on reproductive traits.

4.4.3.3. Number of pods per plant

At 55 days after seeding, the number of pods was determined in each environment, using the same 5 plants in each experimental unit.

4.4.4.4. Grain yield

In addition, grain yield was measured at physiological maturity by harvesting all pods from each experimental unit. After manual threshing, the grains were cleaned and then dried to a uniform moisture content before weighing. While the exact moisture content at weighing was not measured, we followed a standard protocol for beans; as reported by [49] for *Phaseolus vulgaris*. In our case, we operated the drying at 35 °C until constant mass was achieved. The total grain mass per unit was then extrapolated to yield and expressed in tons per hectare (t·ha^−1^). For each genotype and environmental condition, all the 6 experimental units were evaluated, resulting in a total of 24 yield observations.

4.4.4.5. Harvest Index (HI)

For a total of 24 full unit-level harvests, all above-ground biomass and grain were collected from each of the six experimental units at physiological maturity, according to genotype and environmental condition (Control and OTC). All plant material was oven-dried at 65 ± 2 °C to constant weight. Grains were previously separated from the biomass and dried to a uniform moisture content. For each experimental unit, HI was calculated according to the adjusted formula:

$$\mathrm{HI}\;=\;\frac{\mathrm{Grain}\;\mathrm{dry}\;\mathrm{weight}\;(g)}{\mathrm{Grain}\;\mathrm{dry}\;\mathrm{weight}\;\left(g\right)\;+\mathrm{Shoot}\;\mathrm{dry}\;\mathrm{weight}\;}$$

This method ensures that the grain mass is not double counted in the denominator. A total of 24 independent HI values were obtained. The procedure followed the approach described by [50] who used dry weights per plant to calculate HI under different water regimes in common bean.

### 4.5. Leaf Physiological Nutrient Content Under Induced Passive Heat (OTC)

Fully expanded leaves were sampled at the reproductive stage from plants grown under induced passive heat using OTC and from concurrent control plots. Samples were rinsed with distilled water, oven-dried at 60 °C tom constant weight, and ground to a fine powder; particle size was controlled to be in the 0.25–0.42 mm range (we used 250 µm, within the commonly reported <0.42 mm for leaf tissue) [51,52].

Approximately 0.3 g of dry tissue was microwave-digested with 5 mL HNO_3_ (conc.) and 3 mL H_2_O_2_ (conc.); digests were brought to 50 mL with deionized water and analyzed for macronutrients (C, N, P, K, Ca, Mg, S) and micronutrients (Fe, Cu, Zn, Mn, B, Na) by ICP-OES following our laboratory standard operating (LSO) procedure, which specifies calibration levels, wavelengths, and blanks/standards (multi-element GPI-14) [53]. Nutrient contents are reported as % (macronutrients) and ppm (micronutrients). Instrument calibration employed three standards plus a blank; a laboratory control sample was included for drift/precision checks as prescribed in our LSO.

Each value represented one composite analytical sample per environment (OTC or Control) obtained by pooling fully expanded leaves from multiple plants; no plot level biological replication was performed, and no statistical analysis is presented.

### 4.6. Statistical Analysis

The data for the morphological, yield and yield components variables were analyzed using the GLM procedure of the statistical program Minitab Software version 16. The model is characterized by two fixed factors, namely Genotypes (G) (*Tayní* and *Matambú*) and Environment (E) (Control and OTC) as well as their interaction ‘G × E’. They were treated as fixed factors because of the objective of this study. The Tukey test was used to check for significant differences between the treatment means. If *p* < 0.05, the effects and interactions were considered significant. Data were examined for normality before being analyzed, and log transformation was employed to correct them. The abiotic variables were analyzed using a repeated measure analysis of variance (ANOVA). They were compared between the OTC and control environments and summarized for each SensorPush. The correlations between the abiotic variables and the morphological, physiological, and yield parameters were conducted also in Minitab.

## 5. Conclusions

This study demonstrated that Open Top Chambers (OTC) effectively reproduced projected warming for the humid tropics of Central America, increasing daily maximum, minimum, and mean air temperatures by +2.29, +0.93, and +2.80 °C, respectively. Both local bean varieties, *Matambú* and *Tayní*, showed strong declines in yield formation and final yield under induced passive heat; grain yield fell by more than 80% relative to the control. Compared with vegetative growth, yield and its components were more affected by the induced passive heat.

Under induced passive heat, *Matambú* maintained higher nodulation and above-ground biomass than *Tayní*. Across genotypes, root traits increased in OTC (greater fresh weight and length), indicating a shift in allocation under heat. In the correlation analysis, harvest index and pod number showed the strongest associations with yield across environments.

Overall, this field experiment documents the response of two local common bean varieties to induced passive heat simulated with OTC in Costa Rica. The results support the conservation and systematic evaluation of locally adapted materials, such as *Matambú*, to guide selection for improved performance under rising temperatures.

## Figures and Tables

**Table 1:** Main effects of genotype and induced passive heating (OTC) on vegetative growth, biomass accumulation, yield and yield components in two local common bean varieties, in the absence of significant Genotype × Environment interaction.

Variables		Growth and Development			Biomass Accumulation		Yield and Components			
Factor		Plant Height (cm)	Plant Stem (mm)	NLP	RFW (g)	RL (cm)	Yield (t ha^−1^)	PW (cm)	NGP	HI
G	*Matambú*	47.18 a	3.09 a	7.06 a	5.97 a	22.71 a	0.11 a	0.85 a	2.74 a	0.19 a
	*Tayní*	32.12 b	3.00 a	5.85 b	5.08 b	21.63 a	0.08 a	0.76 b	2.43 a	0.12 a
	LSD	0.38	0.03	0.19	0.16	0.05	0.02	0.11	0.12	0.04
E	OTC	36.73 b	2.92 b	7.01 a	6.64 a	23.6 a	0.03 b	0.77 a	2.64 a	0.07 b
	Control	41.26 a	3.19 a	5.89 b	4.57 b	20.81 b	0.15 a	0.84 a	2.51 a	0.23 a
	LSD	0.12	0.08	0.17	0.37	0.12	0.11	0.08	0.05	0.04

G: Genotype; E: Environment; OTC: Open Top Chamber; NLP: Number of Leaf per Plant; RFW: Roots Fresh Weight; RL: Roots length; PW: Pods Weight; NGP: Number of Grain per Pod; HI: Harvest Index; LSD: Least Significant Difference. *Average followed by different letters in the same column were significant at the Tukey Test at p < 0.05*.

**Table 2:** Pearson correlation coefficients among thermal, morphological, physiological, and yield-related variables in two local bean varieties under control and induced passive heat conditions.

	PW	PL	NGP	Yield	HI	SFW	SDW	RFW	RL	NNP	PH	PS	NLP	NPoP	Tmax	Tmin
PL	0.19															
NGP	−0.03	0.80														
Yield	0.25	0.07	−0.08													
HI	0.32	0.110	−0.06	0.94												
SFW	−0.32	−0.13	0.05	−0.93	−0.99											
SDW	−0.24	−0.20	−0.07	0.02	−0.30	0.35										
RFW	−0.32	−0.03	0.08	−0.94	−0.97	0.95	0.16									
RL	0.35	0.03	−0.08	0.90	0.96	−0.94	−0.25	−0.99								
NNP	−0.09	−0.33	−0.09	−0.18	−0.33	0.41	0.67	0.09	−0.11							
PH	0.04	−0.21	−0.13	0.09	0.11	−0.08	0.01	−0.18	0.20	0.26						
PS	−0.12	0.02	−0.03	0.06	0.01	−0.02	0.09	0.04	−0.06	−0.15	0.31					
NLP	0.16	−0.12	−0.20	0.22	0.20	−0.19	0.03	−0.23	0.23	0.06	0.57	0.62				
NPoP	0.08	−0.07	−0.08	0.55	0.45	−0.44	0.12	−0.44	0.40	−0.09	0.19	0.40	0.32			
Tmax	−0.19	−0.07	0.07	−0.98	−0.87	0.86	−0.16	0.86	−0.81	0.18	−0.04	−0.10	−0.20	−0.53		
Tmin	−0.19	−0.07	0.07	−0.98	−0.87	0.86	−0.16	0.86	−0.81	0.18	−0.04	−0.10	−0.20	−0.53	1.00	
Tmean	−0.19	−0.07	0.07	−0.98	−0.87	0.86	−0.16	0.86	−0.81	0.18	−0.04	−0.10	−0.20	−0.53	1.00	1.00

PW: Pod Weight; PL: Pods Length; NGP: Number of Grain per Pod; HI: Harvest Index; SFW: Shoot Fresh Weight; SDW: Shoot Dry Weight; RFW: Root Fresh weight; RL: Root Length; NNP: Number of Nodules per Plant; PH: Plant Height; PS: Plant Stem; NLP: Number of Leaves per Plant; NPoP: Number of Pods per Plant; Tmax: Daily maximum Temperature; Tmin; Daily minimum Temperature; Tmean: Daily medium Temperature.

## Data Availability

The original contributions presented in this study are included in the article/Supplementary Material. Further inquiries can be directed to the corresponding authors.

## Supplementary Materials

The following supporting information can be downloaded at: https://www.mdpi.com/article/10.3390/plants14223489/s1, Table S1: Analysis of variance (ANOVA) for growth, biomass, and yield components evaluated variables of two local bean varieties (*Phaseolus vulgaris* L.) under different environment (Control vs. OTC). Table S2: Soil Chemical Analysis (Lab Report: Sample ID 250776—LEG 02, 17/06/2025).
