# Supplementary material for: Responses to Induced Passive Heat in Two Local Common Bean (Phaseolus vulgaris L.) Varieties Under Humid Tropical Field Conditions in Costa Rica
Source: Plants (Basel). 2025 Nov 15;14(22):3489. doi: 10.3390/plants14223489 (PMC12656148; doi:10.3390/plants14223489)
Supplement: Supplementary file 1 [file plants-14-03489-s001.zip › plants-3926350-supplementary.pdf]

Table S1. *Analysis of variance (ANOVA) for growth, biomass, and yield components evaluated variables of two local bean varieties (Phaseolus vulgaris* L.*) under different environment (Control vs. OTC)*

Variables	Factor	df	MS	F-value	p-value	CV (%)
**Growth and Development**						
Plant height (cm)	G	1	85.42	5.48	0.019*	9.8
	E	1	312.36	22.51	<0.001***	
	G×E	1	47.85	3.88	0.051ns	
Stem diameter (mm)	G	1	0.008	3.12	0.087ns	6.3
	E	1	0.014	5.74	0.017*	
	G×E	1	0.002	1.09	0.32ns	
Number of leaves	G	1	25.18	4.76	0.031*	12.5
	E	1	51.32	9.87	0.002**	
	G×E	1	7.05	1.23	0.27ns	
**Biomass Accumulation**						
Root fresh weight (g)	G	1	5.81	4.12	0.045*	10.8
	E	1	9.73	6.89	0.011*	
	G×E	1	2.64	1.92	0.17ns	
Root dry weight (g)	G	1	0.54	4.95	0.029*	9.6
	E	1	1.12	10.15	0.002**	
	G×E	1	0.28	2.37	0.13ns	
Root length (cm)	G	1	6.18	5.24	0.026*	8.2
	E	1	9.64	8.16	0.006**	
	G×E	1	1.73	1.12	0.29ns	
**Yield and Components**						
Pods per plant	G	1	14.32	6.45	0.015*	11.2
	E	1	22.86	10.02	0.002**	
	G×E	1	4.73	2.11	0.15ns	
Pod width (cm)	G	1	0.012	4.51	0.034*	7.4
	E	1	0.018	6.79	0.011*	
	G×E	1	0.005	1.88	0.18ns	
Pod length (cm)	G	1	0.36	4.18	0.043*	8.6
	E	1	0.72	8.64	0.004**	
	G×E	1	0.18	2.31	0.14ns	
Grains per pod	G	1	1.21	6.12	0.016*	10.9
	E	1	2.07	10.46	0.002**	
	G×E	1	0.41	2.07	0.16ns	
Grain yield (t ha^{-1})	G	1	0.0024	5.01	0.028*	14.8
	E	1	0.0056	11.24	0.001**	
	G×E	1	0.0008	1.43	0.24ns	
Harvest index (HI)	G	1	0.016	6.85	0.011*	9.3
	E	1	0.024	10.14	0.002**	
	G×E	1	0.008	2.74	0.11ns	

G: Genotype; E: Environment; df: Degree of freedom; MS: Mean Square; CV: Coefficient of variance.

Table S2. *Soil Chemical Analysis (Lab Report: Sample ID 250776 – LEG 02, 17/06/2025).*

Parameter	Unit	Value
pH (H_2O)		4.79
Extractable Acidity	cmol(+)·kg^{-1}	1.90
K (exchangeable)	cmol(+)·kg^{-1}	0.23
Ca (exchangeable)	cmol(+)·kg^{-1}	2.73
Mg (exchangeable)	cmol(+)·kg^{-1}	0.88
Na (exchangeable)	cmol(+)·kg^{-1}	0.74
P (available)	ppm (mg·kg^{-1})	1.5
Fe (DTPA/Mehlich)	ppm (mg·kg^{-1})	68
Cu	ppm (mg·kg^{-1})	2.4
Zn	ppm (mg·kg^{-1})	0.2
Mn	ppm (mg·kg^{-1})	45
B	ppm (mg·kg^{-1})	0.7
S	ppm (mg·kg^{-1})	65
